# Editorial: Aging-related factors in digital health: Design, uptake, engagement, and outcomes

**DOI:** 10.3389/fdgth.2022.1124464

**Published:** 2023-01-09

**Authors:** OraLee H. Branch, Sarah A. Graham, Raeanne C. Moore, Patricia A. Areán

**Affiliations:** ^1^ Department of Clinical Research, Lark Health, Mountain View, CA, United States; ^2^ Department of Psychiatry, School of Medicine, University of California, San Diego, La Jolla, CA, United States; ^3^ Department of Psychiatry and Behavioral Sciences, School of Medicine, University of Washington, Seattle, WA, United States

**Keywords:** mhealth, patient activation, preventive health, mental health, usability, acceptability, adherence

**Editorial on the Research Topic** Aging-related factors in digital health: design, uptake, engagement, and outcomes

This Research Topic explores digital health technologies designed to facilitate chronic disease prevention and management for older adults. Adults over age 65 have a high prevalence of chronic diseases, with 86% having at least one chronic condition such as diabetes or hypertension (1) and ∼12% reporting subjective cognitive decline (2). These conditions are costly: in recent estimates, despite accounting for only 15% of the US population, older adults accounted for 34% of total healthcare expenditures (3). As society ages (4) and provider availability and time dwindle (5), enhancing older adults' ability to effectively self-manage their health is paramount.

For patients to successfully manage their health, they must exhibit “patient activation”—actively demonstrating the willingness and ability to take independent actions to manage their health and care (6). Digital health technologies offer a scalable means to facilitate patient activation by increasing patient knowledge, equipping patients with self-management techniques, and offering feedback and support to improve patient confidence.

The articles in this research topic collection address three central themes: (1) Older adults are willing and able to use digital health technologies when provided the opportunity, (2) They exhibit excellent adherence when using these technologies, and (3) Their use of these technologies generates unique, actionable health information and results in positive health outcomes.

Despite the potential of digital health technologies, skepticism exists regarding their utility for older adults. A considerable body of research has focused on the “Digital Divide” as it pertains to age-related willingness and ability to use digital health technologies (7). However, older adults' use of technology continues to increase, and the notion that older adults are unwilling or incapable of using technology is outdated.

As of 2021, 61% of older adult Americans owned a smartphone, representing a considerable increase from 27% in 2015 (8). The gaps between the oldest and youngest adults have narrowed in areas such as smartphone and tablet computer ownership and social media and internet use, with 75% of older adults now reporting internet use (8). These trends are supported by papers in this special issue by Graham et al. and Auster-Gussman et al. Older adults do engage with and have success when using technologies that require smartphone and internet use when provided with opportunities from their healthcare provider to engage in digital preventive health programs. In fact, engagement among older adults exceeded that of younger adults (Graham et al.), and older adults experienced beneficial outcomes such as weight loss while using these programs (Auster-Gussman et al.). Although contrary to outdated assumptions that older adults struggle to use technology, the high engagement and positive outcomes are consistent with the data described throughout this Research Topic Collection. Older adults may represent a group that takes full advantage of opportunities to better self-manage their health when presented with digital technologies.

Digital technologies can provide greater access to personalized health information and generate key insights that can improve care quality and activate patients to achieve better health outcomes. Quality care and improved outcomes benefit all ages, but older adults stand to benefit from digital health technologies to the greatest extent given the complexity of managing multiple health conditions simultaneously, the need for frequent and real-time care, the need to understand the dynamics of older adult behaviors in a home environment, and challenges to adhering to care management recommendations introduced by the normal aging process.

In this collection, Paolillo et al. demonstrate that older adults are willing and able to adhere to a wearable device (Fitbit) protocol that enables accurate monitoring of physical activity behaviors. Accuracy in measuring physical activity behaviors is critical in evaluating the effectiveness of lifestyle interventions, and as VandeBuente et al. demonstrate, the accuracy of subjective physical activity reporting decreases with declining memory and executive performance–common consequences of aging. Research demonstrates that wearable devices provide more valid estimates of physical activity behaviors than subjective reporting, providing actionable information to both patients and providers.

To maximize their potential, designers of digital technologies must understand the fundamentals of aging and incorporate age-related considerations (e.g., motor, perceptual, and cognitive capabilities) into the design of their products. Research must continue to focus on older adults' adoption of these technologies, engagement, and perceptions of usefulness.

In this collection, Badal et al., Klaus et al., and Moore et al. demonstrate that surveys deployed *via* smartphone technology such as ecological momentary assessment (EMA) and EMA along with mobile cognitive testing are feasible and acceptable to older adults and present unique opportunities for assessment of health. Use of smartphone technology is a valid way to collect real-time behaviors, experiences, and emotions without reliance on retrospective recall or influence of current mood state. The ability to serially collect data in the individual's environment revealed relationships among negative affect, loneliness, and adaptive behaviors (Badal et al.). EMA resulted in high adherence (Klaus et al.), even when older adults had potential barriers to engagement such as mild cognitive impairment (Moore et al.). Reaching the older adult population during the COVID-19 pandemic to facilitate the maintenance of health and well-being was of critical importance, and EMA offered a remote and scalable means to do so.

Digital health technologies enable data collection and patient activation in the natural environment. In addition to 24/7 tracking of daily behaviors and experiences, digital technologies can also support essential round-the-clock aspects of care management such as medication adherence. In this collection, Gualtiere et al. describe how medication storage location impacts adherence and discuss how technology can play an important role in promoting adherence, which can also be impacted by things like worsening memory with age.

When deploying digital health technologies, consideration must be given to older adults' perceptions and attitudes toward these technologies. In this collection, Woerner et al. observed that although digital technologies for mental health are considered valuable by all age groups, older adults prefer that they play a complementary or supportive role rather than primary role in their care. Continued research and collaborations between industry, where products are designed, built, and maintained, and academic institutions, where research designs can be optimized, should be encouraged to best reveal how digital health technologies should be deployed and leveraged in practice for older adults.

Digital health technologies represent a scalable and cost-effective opportunity to activate older adults in self-managing their health and care. As demonstrated in this special issue of Frontiers in Digital Health, older adults are willing to use digital health technologies when provided the opportunity, they exhibit excellent adherence when using these technologies, and their use of these technologies generates actionable health information and positive results. Best practices for optimizing the older adult user experience and the implementation of these technologies within the healthcare environment should remain a focus of future research, including patterns of digital health use across racially and ethnically diverse older populations.
